# Outpatient referrers as a pathway to care for a new family-centered health intervention in psychiatric clinics and how to reach them: evaluation of an implementation strategy

**DOI:** 10.1186/s12913-025-13031-x

**Published:** 2025-07-01

**Authors:** Philip Martin Kaczmarek, Carolin Laser, Silke Wiegand-Grefe, Silke Pawils

**Affiliations:** 1 Institute for Epidemiology, Social Medicine and Health System Research, Hannover Medical School, Carl-Neuberg-Straße 1, Hanover, 30625 Germany; 2 Center for Psychosocial Medicine, Department of Medical Psychology, University Medical Center Hamburg-Eppendorf, Martinistraße 52, Hamburg, 20246 Germany; 3 Department of Psychiatry and Psychotherapy, University Medical Center Hamburg-Eppendorf, Martinistraße 52, Hamburg, 20246 Germany; 4 German Center for Child and Adolescent Health (DZKJ), partner site Hamburg, Martinistr. 52, Hamburg, 20246 Deutschland

**Keywords:** Pathway to care, Gatekeeper, Regional care, Outpatient care, Dissemination of information, Children of mentally ill parents, Implementation research

## Abstract

**Background:**

The integration of evidence-based research into clinical practice faces several challenges. One major barrier is the effective dissemination of information regarding new interventions. To illustrate this, the CHIMPS-NET (Children of Mentally Ill Parents) program, a family-centered intervention in German psychiatric clinics, is used as an example. This study evaluates the referral criteria, the dissemination of information, and the accessibility of outpatient specialists involved in the gatekeeping process for accessing and referring patients to CHIMPS-NET. This study serves as part of the implementation strategy for the sustainable anchoring of the CHIMPS-NET program.

**Methods:**

We identified all outpatient professionals (*n*=2828) in each catchment area of the psychiatric specialist clinics that provided the new CHIMPS-NET form of care. We identified them as potential gatekeepers. These professionals worked across a range of fields, some had a psychological professional background. Professionals in the intervention group (*n*=946) received information from us about the new intervention, while professionals in the control group (*n*=1882) did not. Before and after the information was sent by post, all participants were asked via a questionnaire about their knowledge of family-centered intervention and its importance for the vulnerable target group of children of mentally ill parents. There was an interval of three months between the first questionnaire and the information being sent out and a further three months after this the final questionnaire was sent.

**Results:**

There was no significant change in knowledge across the two measurement points. Reaching out to gatekeepers had no influence on knowledge of the CHIMPS-Net intervention. However, awareness of family-centered health interventions was four times higher if the gatekeeper had a psychological professional background.

**Conclusion:**

Dissemination of information by post about CHIMPS-Net was not effective at communicating information or reaching gatekeepers. Knowledge of family-centered health interventions was four times higher among the group of specialists and therapists with a psychological background. We therefore suggest that general (somatic) practitioners, who make up most of the gatekeeper population in Germany, need to be approached more intensively than professionals with a psychological/psychiatric background. It is also recommended that individual clinics address their catchment area more intensively and directly.

**Supplementary Information:**

The online version contains supplementary material available at 10.1186/s12913-025-13031-x.

## Contribution to literature

- To the best of our knowledge, the present study represents the first attempt to consider outpatient gatekeepers during the implementation process of an evidence-based inpatient family intervention (CHIMPS-Net). It aimed not only to inform them about the intervention but also to engage them in their role as referrers to provide the best possible care for the patient group at risk.
- The study highlighted that outpatient care professionals are difficult to reach with information about inpatient interventions in their care region, even when provided with such information, emphasizing the need for improvement.

## Background

For years, implementation science has been working on the sustainable integration of evidence from research into routine care [1, 2]. It describes, organizes and helps us to understand the complexity of integrating these research findings into healthcare systems and provides criteria and models that should be considered during the development of evaluation design [3, 4]. Nevertheless, in the majority of cases, it is not possible to ensure the transfer of research results to care practice. There are many reasons for this, with one being how well information about the existence and value of research findings is passed on to the professionals concerned [3, 5]

The *CHIMPS-NET* (Children of Mentally Ill Parents Network) focuses on the care needs of children of mentally ill parents as a more vulnerable target group. Their needs are often overlooked in the treatment of their mentally ill parents in psychiatric institutions [6]. This disregard can have long-term, lifelong consequences for children, including an increased risk of mental disorders and developmental problems [7–9]*.* To improve the treatment of mental illness in this vulnerable target group, *CHIMPS-NET* aims to meet the specific needs of these children and their parents using preventative measures and treatment in group and individual family therapy settings [10]. During project planning for the *CHIMPS-NET* evaluation project there was already a strong focus on ensuring the best possible implementation strategy for the sustainable anchoring of the program [11]. The implementation project was therefore accompanied by 1) the establishment of screening instruments, also within psychiatric centers, for the target patient group at risk (evaluated in a previous study* -* see study protocol, [11]), 2) training of professionals within adult psychiatric centers and 3) gatekeepers for outpatient referrals to be informed for optimal pathways to care (current study at hand) [12, 13]. Gatekeeping means that patients in Germany generally visit a primary care provider first, who decides whether specialist care is needed. Such a referral regulates access to specialist care, hospital treatment or diagnostic tests. The aim of this system is to better control healthcare costs and ensure more targeted and efficient hospital care [14]. Gatekeepers use specific criteria to determine the need for inpatient psychiatric treatment when making recommendations. Individual patient criteria are taken into account in addition to the care region (sectoralization). Sectoralization in the German healthcare system refers to the division of the healthcare system into various specialized sectors that provide different types of medical services. This structure leads to a separation between outpatient and inpatient care, as well as different specialties within the healthcare system. It also includes the concept of catchment areas. This refers to the division of healthcare services not only by type (outpatient vs. inpatient) but also by geographic location. Each healthcare provider or institution typically serves a specific region or population, with varying access to resources depending on the location. The involvement of gatekeepers in the process of implementing interventions such as the *CHIMPS-NET* can strengthen collaboration between outpatient and inpatient care and improve overall patient care [13].

Wahlert and Mestel [15] list three categories of indication criteria: medical indication criteria, indication criteria according to treatment need and individual indication criteria. The medical indication criteria include the severity of the illness and the risk to oneself and others. The indication criteria according to treatment needs refer to chronicity and complexity. The individual indication criteria consider demographic characteristics and underage children. Gatekeepers therefore have an important interface function between the outpatient, the external environment and the internal structures of psychiatric care [16, 17].

The implementation strategy for informing gatekeepers is evaluated using the example of the *CHIMPS-NET* research project on preventive care for children of mentally ill parents. *CHIMPS-NET*, a 4-year (Jan. 2020 to Dec. 2023) innovation fund project of the Joint Federal Committee of Physicians and Health Insurance Funds in Germany, provides the methodological framework for the present study as a model project. The prevention and therapy program (*CHIMPS-NET*) for children and adolescents (0–21 years) with mentally ill parents was introduced in 18 adult psychiatric clinics in Germany with the aim of improving the mental health and quality of life of the families. The *CHIMPS-NET wa*s geared toward the individual needs of families and combines psychoanalytic family therapy with systemic and psychoeducational content [18].

**Fig. 1:**
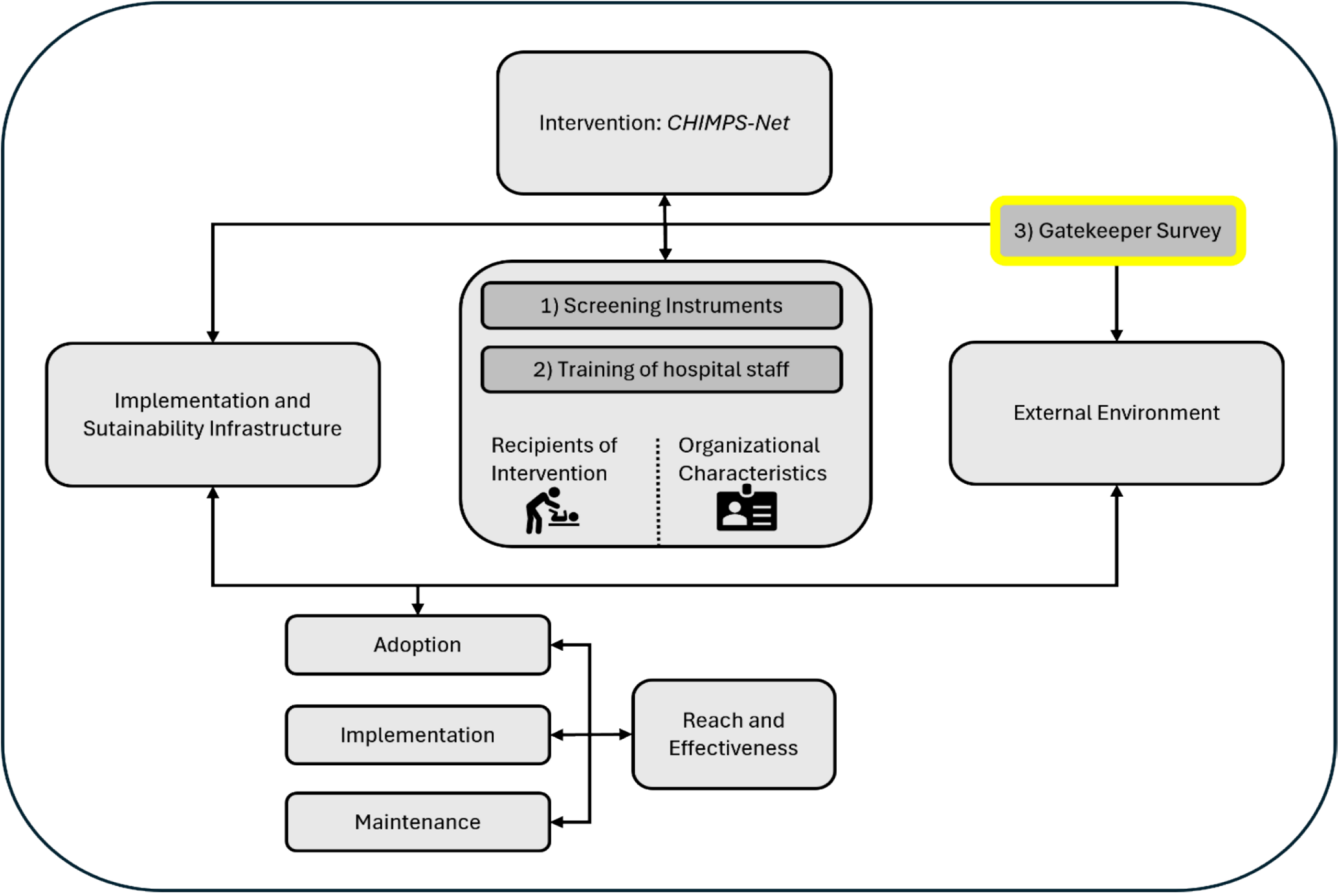
Adapted *PRISM (Practical, Robust Implementation and Sustainability Model)* (based on [3]). This adapted prism diagram shows the relative role of the three implementation studies: 1) screening instruments, 2) training hospital staff and 3) the Gatekeeper Survey (current study at hand)

In terms of an implementation strategy for *CHIMPS-NET,* the *PRISM* model (Practical, Robust Implementation and Sustainability Model, Fig. 1) is suggested as a framework for assessing and promoting the sustainable implementation of interventions in the care region [3]. In particular, the explicit assessment of the implementation and sustainability of infrastructure and the external environment should be emphasized. As an essential goal of implementation, it can be deduced that the communication between and information held by all the actors involved—in this case, the gatekeepers—is important [3, 19]. The existence of offerings such as *CHIMPS-NET* is not of itself sufficient if the gatekeepers are in the first instance not aware of the program or are difficult to reach in order to disseminate information and thus make them newly aware. This makes it significantly harder for patients to access the programme.

Participating adult psychiatric clinics (*n*=18) were cluster-randomized to intervention (IG) or control group (CG) during the assessment of the CHIMPS-Net study of which this study at hand is a component. The intervention clinics were supported by three implementation projects [11]: a) establishing screening instruments to identify affected families [20]; b) training clinical professionals to strengthen attitudes, knowledge and skills [21]; and c) dissemination of information/gatekeeper survey (current study).

The questions for evaluating the implementation in the study at hand are as follows.

1. What knowledge do gatekeepers have about the prevalence of underage children living in the same household as their mentally ill patients? Does this knowledge vary according to professional background?
2. Does a specific dissemination of information to gatekeepers change their knowledge of the family-oriented care offered by the *CHIMPS-NET* and their ranking of the referral criteria?

## Method

### Sample characteristics

All outpatient general practitioners and specialists, psychiatrists, and neurologists in the psychiatric care region of the participating *CHIMPS-NET* clinics that are eligible to refer patients for inpatient care were defined and included as gatekeepers. Additionally, we only included professionals that treat adults. We defined sectoralized psychiatric care as the legal obligation of the hospital to admit and care for mentally ill people from a particular geographical region (care region) [22]. All specialist clinics provided their compulsory care regions and zip codes. On this basis, the search for practitioners was carried out online via the associations of statutory health insurance physicians in the federal states. Gatekeepers with multiple qualifications were also included. In addition, 245 psychiatric outpatient specialist addresses within the care regions were acquired through the address provider *Scitrace.* A sample of *N*=2,828 professionals from professional groups in 18 care regions in Germany was compiled. The contact details (postal address and/or e-mail address) were recorded, and multiple entries were excluded. A sample of general practitioners (family doctors, internists, general practitioners), psychiatrists (neurologists, psychiatrists) and psychotherapists (medical and non-medical) from 18 care regions in Germany was compiled, and multiple entries were excluded. The exclusion criteria were all gatekeepers who were not located in the care region defined by the specialist clinic at the time of the survey or who could not be specifically assigned to one of the above-mentioned professional qualifications. In addition, nonparticipants were asked about their reasons for not taking part.

Overall, for t0 *n*=1882 there were subjects in the control group of which *n*=221 participated, and *n*=946 participants in the intervention group of which *n*=88 participated.

### Operationalization and instruments

In the following, if an email address could be found, test subjects in this sample were surveyed using a semi-structured online questionnaire developed by the *SoSci-Survey* web application for creating online questionnaires. If no email address could be determined, the respondents were surveyed by post using a semi-structured paper-pencil questionnaire. The questions used in both questionnaires were identical. Of a total of 2828 respondents, 1362 were contacted by e-mail, and 1466 were contacted by post. First, all of the sample group were asked whether they referred patients to the regional psychiatric clinic and the average number of patients referred per year. In addition, the sample group were asked about the following constructs: 1) importance of certain referral criteria, 2) knowledge and awareness of children of mentally ill parents, and 3) sociodemographic (Table 1; For detailed information, see the supplementary data/supporting information). Gatekeepers’ awareness of internal clinic services for parents with underage children was operationalized as a binary measure, where respondents answered “yes” or “no” to indicate their recognition of the existence of such services. If subjects decided not to participate in the study, they were able to give reasons for their decision in the “nonparticipant survey”: 1) no interest, 2) no time, 3) bad experience with surveys, and 4) other.

**Table 1:** Sociodemographics of the gatekeepers (*N*=512)

Category	Total	t0 n (%)		t1 n (%)	
		Intervention *n*=88	Control *n*=221	Intervention *n*=69	Control *n*=134
Gender					
Female	257 (50,9%)	49 (55,7%)	111 (50,7%)	36 (53,7%)	61 (46,6%)
Male	247 (48,9%)	39 (44,3%)	108 (49,3%)	30 (44,8%)	70 (53,4%)
Miscellaneous	1 (0,2%)	0		0	
Profession					
Psychiatrist	64 (12,5%)	14 (15,9%)	23 (10,5%)	11 (16,4%)	16 (12,2%)
Neurologist	34 (6,6%)	4 (4,5%)	13 (5,9%)	6 (9,0%)	11 (8,4%)
Neurology	8 (1,6%)	0	3 (1,4%)	1 (1,5%)	4 (3,1%)
Familydoctor/general practitioner/internist/practicing physician	379 (74,0%)	66 (75%)	172 (78,2%)	45 (67,2%)	96 (73,3%)
Medical psychotherapist	41 (8,0%)	4 (4,5%)	13 (5,9%)	11 (16,4%)	13 (9,9%)
Specialistinpsychosomatic medicine and psychotherapy	11 (2,1%)	1 (1,1%)	8 (3,6%)	0	2 (1,5%)
Miscellaneous	12 (2,4%)	3 (3,4%)	6 (2,7%)	1 (1,5%)	2 (1,5%)
Type of practice					
Individual practice	331 (65,9%)	51 (58,6%)	147 (67,4%)	41 (62,1%)	92 (70,2%)
Joint practice	148 (29,5%)	32 (36,8%)	62 (28,4%)	23 (34,8%)	31 (23,7%)
MVZ	23 (4,6%)	4 (4,6%)	9 (4,1%)	2 (3,0%)	8 (6,1%)
Cash register seat					
Legal	5 (1,0%)	0	1 (0,5%)	1 (1,5%)	3 (2,3%)
Private	0	0	0	0	0
Statutory & private	503 (98,2%)	88 (100%)	219 (99,5%)	67 (98,5%)	129 (97,7%)
Category	Total n (%)	t0 M (sd) range		t1M (sd) range	
		Intervention *n*=88	Control *n*=221	Intervention *n*=69	Control *n*=134
Age	56,22 (9,5) 34 - 86	56,04 (10,24) 36 - 80	56,37 (9,63) 34 - 86	55,62 (9,86) 35 - 78	56,39 (8,69) 36 - 80
Duration of employment	26,4 (10,6) 2 - 60	26,07 (10,88) 3 - 51	26,31 (11,06) 2 - 60	25,98 (10,21) 4 - 50	26,98 (10,01) 3 - 55

### Intervention

**Fig. 2:**
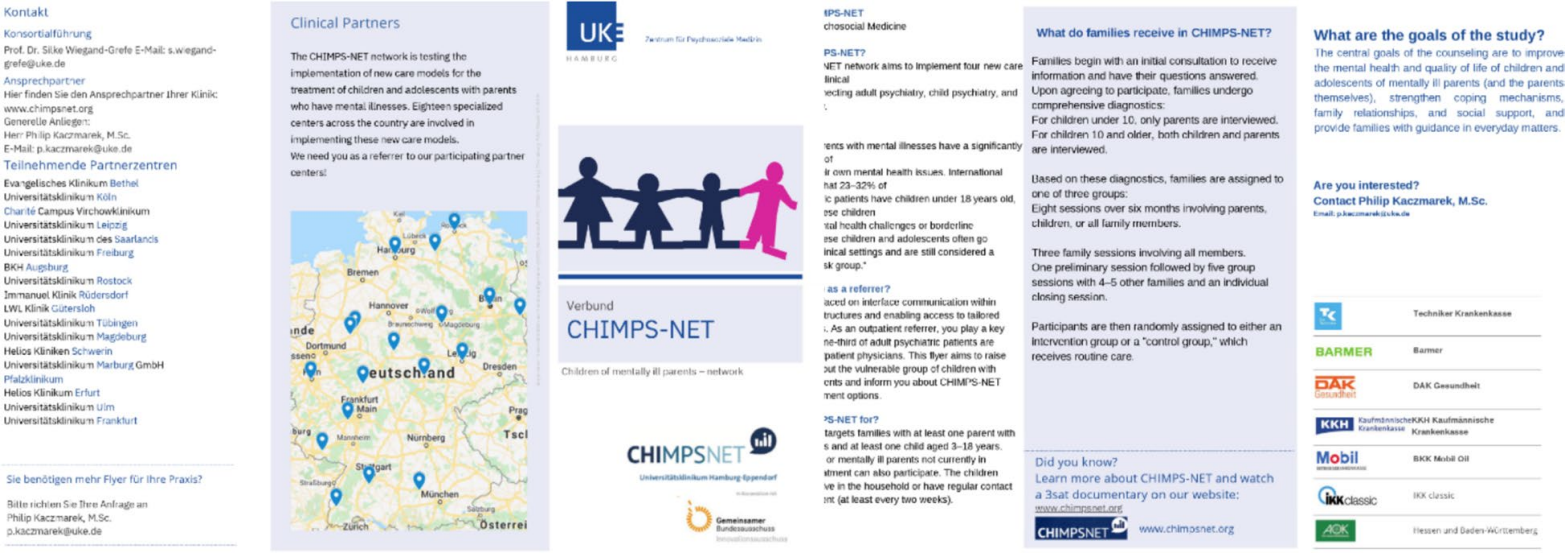
*CHIMPS-NET* information flyer (front and back)

The intervention used a flyer providing information about the target group, the content and the objectives of the new *CHIMPS-NET* form of care. This information flyer was sent by post - to a named individual - to the intervention group consisting of *n*=946 subjects 3 months after t_0_; the control group, consisting of 1882 subjects, did not receive an information flyer. The focus of the flyer is on emphasizing interface communication between the various care structures. Accordingly, gatekeepers are informed about the regional treatment options of a specialist psychiatric clinic with the new *CHIMPS-NET* form of care. The flyer headings include “What is *CHIMPS-NET*?”, “Necessity” and “What can I do as a gatekeeper?” (see Figure 2).

One month after the flyer dissemination, the follow-up measurement (t_1_) took place, which was delivered the same way as t0, reaching out to *n*=2828 subjects from both groups. This time, out of *n*=1882 subjects of the control group, *n*=134 participated. Out of *n*=946 subjects in the intervention group, *n*=69 participated.

### Data evaluation

The evaluation is based on the complete survey of the regional gatekeepers of the 18 participating adult psychiatric clinics. The data of the gatekeepers who participated in the survey at time points t_0_ (baseline) and t_1_ (follow-up, three months later) were assumed to be homogeneous. The data were analyzed using IBM SPSS Statistics (version 25). To test whether the mean values of the two independent samples differed from each other, single-factor analyses of variance or t tests were performed. According to Cohen [23], the limits for the size of the effect are.01 (small effect),.06 (medium effect) and.14 (large effect). The Pearson chi-square test was used to test whether the empirically observed distribution of the categorical variables differed from a certain hypothetically assumed distribution. Effect sizes are interpreted here according to Cohen [23], similar to a correlation. A binomial logistic regression was calculated to examine the extent to which the factors of professional affiliation (*psych (*profession group specialists in psychiatry-neurology-psychosomatics) *vs. soma (*family doctor/general practitioner/internist/practising physician)), time of survey *(t*_*0*_* vs. t*_*1*_) and group affiliation (*CG vs. IG*) contributed to gatekeepers’ awareness of internal clinic services for parents with underage children. Effect sizes are interpreted here according to the recommendations of Backhaus [24]. The statistical significance for all analyses was determined with a confidence interval of 95%. The analyses mentioned are based on aggregated data. In this study, missing data were excluded to ensure the completeness of the dataset used for analysis. No additional preprocessing steps were applied to modify or transform the remaining data, preserving the natural variability within the dataset. A power analysis was not conducted prior to the study.

## Results

### Sociodemographics of the sample

In total, and across both survey times t_0_ and t_1_, 512 completed questionnaires were available for evaluation, which corresponds to an overall response rate of 18.1% with 2828 questionnaires sent out. Due to the anonymous nature of the questionnaires it is not possible to state the number of unique participants or how many completed both the t_0_ and t_1_ questionnaires. At time t_0_, *N*=309 (11%), and at time t_1,_*N*=203 (7.2%) questionnaires were available for evaluation. Participation in the online survey was *n*=59 (11.5%), and participation in the postal survey was *n*=453 (88.5%). The response rates of the respective hospital sites differed significantly in some cases, with responses ranging from 6.9% (Charité Campus Mitte Berlin) to 42.2% (Rostock University Hospital). 50.9% were female, 48.9% were male, and 0.2% selected the option “diverse”. On average, the respondents were 56.22 years old and had 26.4 years of professional experience. The most common professional groups among the participants were family doctors, general practitioners, and internists (74.0% (*n*=512)). The other professional groups were distributed as follows: psychiatrists (12.5%), neurologists (6.6%), other specialists in neurology (1.6%), medical psychotherapists (8%), specialists in psychosomatic medicine and psychotherapy (2.1%) and others (2.4%). The majority of respondents (*n*=512) worked in individual practices (65.9%) or group practices (29.5%), and almost all (98.2%) treated both patients with statutory health insurance and private patients. Of the participating *N*=512 subjects, *n*=405 (79%) referred patients to the adult psychiatry department of the respective specialist clinic and were therefore, by definition, *gatekeepers*. On average, the respondents referred 9.6 patients per year to adult psychiatry wards (range 0 - 100) (Table 1). A total of *N*=230 of the respondents disagreed to participate in the study. “No time” (57.0%), “Other” (33.9%) and “No interest” (28.3%) were the most frequently cited reasons for nonparticipation.

### Allocation criteria

The questionnaire asked the respondent to rate the allocation criteria on a scale of 1–5. A value of 1 was defined as very unimportant, and 5 was defined as very important. The following frequency distributions were obtained from the 512 responses across the two questionnaires. The *severity of current symptoms* was rated as M=4.90 (sd=0.34), and the *course of disease to date was rated* as M=4.17 (sd=0.73), the most important criteria for allocation. Table 2 lists the least important criteria: *marital status* (M=2.40, sd=1.04) and *age of the patient* (M=2.35, sd=1.06).

**Table 2:** Importance of certain assignment criteria (*N*=512)

Assignment criteria	Missing n	Total M (sd) range	t0 M (sd) range		t1 M (sd) range	
		Intervention *n*=88	Control *n*=221		Intervention *n*=69	Control *n*=134
Severity of the current symptoms	11	4,90 (0,34)	4,84 (0,40)	4,91 (0,28)	4,94 (0,24)	4,90 (0,43)
		1 - 5	3 - 5	4 - 5	4 - 5	1 - 5
Course of the disease to date	13	4,17 (0,73)	4,14 (0,72)	4,16 (0,70)	4,14 (0,89)	4,21 (0,69)
		1 - 5	2 - 5	2 - 5	1 - 5	2 - 5
Medical history	14	3,98	3,98	3,96	3,85	4,07
		(0,82)	(0,85)	(0,79)	(0,90)	(0,82)
		2 - 5	2 - 5	2 - 5	2 - 5	2 - 5
Patient request for inpatient treatment	10	3,83 (0,93)	3,80 (0,96)	3,84 (0,92)	3,80 (0,96)	3,84 (0,94)
		1 - 5	1 - 5	1 - 5	1 - 5	1 - 5
Minor children in the household	27	3,37 (1,03)	3,43 (1,22)	3,33 (1,03)	3,45 (1,00)	3,35 (0,93)
		1 - 5	1 - 5	1 - 5	1 - 5	1 - 5
Professional situation	22	2,83 (0,99)	2,81 (1,10)	2,80 (0,94)	2,99 (0,99)	2,80 (1,01)
		1 - 5	1 - 5	1 - 5	1 - 5	1 - 5
Marital status	19	2,40	2,41	2,33	2,45	2,49
		(1,04)	(1,08)	(0,99)	(1,03)	(1,01)
		1 - 5	1 - 5	1 - 5	1 - 5	1 - 5
Age of the patient	25	2,35	2,45	2,35	2,27	2,34
		(1,06)	(1,15)	(0,98)	(1,12)	(1,10)
		1 - 5	1 - 5	1 - 5	1 - 5	1 - 5

For a statistical mean comparison of the allocation criteria between the intervention and control groups, single factor analyses of variance were conducted at both study time points. There was no statistically significant difference between the intervention groups and the importance of the allocation criterion of having *children in the household*, Welch test *F*(1, 132.92) = 0.39, *p* =.534 at time t_0_. No significant difference was found at time t_1_*, F(1*, 190) = 0.40, *p=* 0.508.

The questionnaire further asked respondents to assign the referral criteria to one of a total of 8 ranks (rank 1 = the most important criterion and rank 8 = the least important criterion). The evaluation of the ranking order of *severity of symptoms* (1st), *previous course of illness* (2nd), *medical history* (3rd), *patient’s wish for inpatient treatment* (4th), *children in the household* (5th), *professional situation* (6th), *marital status* (7th), and *age of the patient* (8th) showed that these did not significantly differ either due to the group affiliation or due to the time of measurement. Single-factor analyses of variance were also carried out for the criterion of having *children in the household.* No significant difference was found between the control and intervention groups at either time point: *F*(1, 285) = 1.28, *p* = 0.261 for t_0_ and *F*(1, 186) = 0.91, *p* = 0.341 for t_1_.

### Knowledge of underage children in the household of mentally ill parents

Participants were asked to estimate the proportion of adult psychiatric patients with a child living in the household (prevalence). On average, the prevalence in both groups was estimated to be 28% (SD=16.57; range 0.00 - 85.00). The estimate of the prevalence of adult psychiatric patients with a minor child living in the household was examined using a t test for mean comparisons in the control and intervention groups. There were no significant differences either for the first survey time point t_0,_*t*(268) = 0.25, or for the second survey time point t_1,_* t*(179) = 0.80.

Furthermore, the estimate of the prevalence of underage children living in the household of a mentally ill parent was compared between the groups of somatic physicians and psychologists and psychiatrists at the time point. A single-factor analysis of variance revealed a significant difference: *F*(1, 443) = 5.69, *p* = 0.017 across both measurement points and in relation to measurement point t_1_: *F*(1, 174) = 5.43, *p* = 0.021.

### Influence of flyer distribution on knowledge about the family-centered form of care

The regression model was statistically significant, χ2(3) = 18.407, *p* <.001, with a low variance explained by Nagelkerke’s [25] R2 =.086, compared to the recommendations of Backhaus et al. (2003). Of the three variables included in the model, occupational affiliation (*p* <.001) was significant, while time (*p* =.371) and group affiliation (*p* =.879) had no significant effect on the predictive performance of the model. Occupational affiliation had a positive effect, with an odds ratio of 4.071 (95% CI [2.079, 7.972]). Consequently, awareness of the services offered to parents is 4.1 times greater if they belong to the professional group *psych* (psychotherapists, specialists in psychiatry-neurology-psychosomatics) than if they belong to the *soma* (family doctor/general practitioner/internist/practicing physician). All the model coefficients and odds values can be found in Table 3.

**Table 3:** Influence of postual information dissemination on knowledge about the CHIMPS-Net

Variables in the Equation								
		B	S.E.	Wald		Exp(B)	95% C.I.for EXP(B)	
							Lower	Upper
Step 1a	Time(1)	0.307	0.343	0.8	0.371	1.359	0.694	2.663
	CG/IG	0.055	0.363	0.023	0.879	1.057	0.518	2.154
	Soma/Psych	1.404	0.343	16.756	<.001	4.071	2.079	7.972
	Constant	−3.174	0.306	107.305	<.001	0.042		

Degrees of freedom were 1 for all Wald statistics

## Discussion

This study evaluated a subproject of a comprehensive implementation strategy for the realization of the evidence-based form of care *CHIMPS-NET*. To ensure the utilization of this form of care in German psychiatric clinics, the referral criteria, information dissemination and the accessibility of outpatient specialists involved in the referral process (gatekeeping) were evaluated.

Knowledge about the vulnerability of the target group - children of mentally ill parents - is present among all gatekeepers, while knowledge about specific, family-centered, regional inpatient care is missing, even when gatekeepers are directly provided with the relevant information. This knowledge-action-gap [26, 27] could not be closed by the provision of written information material (flyers sent by post) and therefore could not contribute to support the implementation of the *CHIMPS-NET* care offer.

The results show that the priority of the allocation criteria with respect to the clinical recommendations (e.g., according to [15]) are consistent. The gatekeepers’ knowledge of this topic is also in line with the findings of current epidemiological studies [12, 28, 29]. However, it was also found that gatekeepers with psychological or psychiatric specialist training estimate this prevalence to be significantly greater than those with purely somatic training. Awareness of the parental services was four times higher if the gatekeeper had a psychological professional background. In addition, those gatekeepers also have greater knowledge reagarding the vulnerable target group of children of mentally ill parents. Hypothetically, the reasons for this range from professional group-specific specialist knowledge, experience, and sensitivity to the topic to differences in diagnostic practice.

The outpatient gatekeepers surveyed were able to assess the prevalence of their adult patients with a minor child according to the literature, and this criterion is adequately considered in relation to other referral criteria.

### Limitations

Nevertheless, limitations must be considered when interpreting the results. The pre-post comparisons were made based on aggregated data without being able to establish individual matching between measurement points. This approach was chosen to avoid having to code the participants and to ensure maximum anonymity of the participants, which however reduced the informative value of the comparison and the validity of the study. Going forward, it is recommended that future research should provide individual matching between measurement points. In addition to this, the skewed allocation between control and intervention group must be acknowledged as a limitation as well as the low response rate which influenced the statistical power and generalizability of results. The differences in participation rates have significant implications for sampling bias and the generalizability of survey results, which are also displayed in participation rates among online vs postal survey respondents. In addition to this we could not monitor whether participants a) received and b) actually read the flyer.

A sample selection of the participants and thus a bias in favor of already interested gatekeepers must be assumed. This bias could lead to an overestimation of the reported knowledge about the prevalence of underage children of mentally ill parents within the target group of gatekeepers.

Furthermore, the conditions and context in which the study was conducted must also be considered. The data collection took place during the COVID-19 pandemic, which could have influenced the receptiveness of the participants, especially regarding the intervention (flyer) to raise awareness of the problems of children of mentally ill parents.

Also, we are aware that information alone is unlikely to change gatekeepers’ behavior. It is also possible that the way the information was presented, the structure of the flyer and the content might also have had either a positive of negative impact.

Given the explorational nature of the paper at hand we are looking forward to additional interventions that are also applicable in this fast-moving and complex area of healthcare.

## Conclusion

Despite the limitations of this study, it can be concluded that gatekeepers are hard to reach, especially for information dissemination regarding new healthcare interventions in regional inpatient care. From a patient perspective, we identify this as a potential entrance barrier to new forms of care. Our study showed that knowledge of family-centered health interventions was four times higher among the group of gatekeepers with a psychological background. However, for many patients, contact with a general (somatic) practitioner is the first point of contact in the German outpatient healthcare system. It should also be noted that it can sometimes take months to get in touch with an outpatient psychiatrist or psychotherapist (*the external environment).* Therefore, general practitioners need to be made more aware of new care services. Information dissemination via flyer has proven to be insufficient to inform gatekeepers about the new form of care, in this example *CHIMPS-Net*. Further and more controlled research is needed. We recommend that communication between clinics and outpatient gatekeepers should be improved by initiating continuous, low threshold contact on a regional basis (*perspective of patients and gatekeepers).*

Finally, it should be noted that the majority of psychiatric inpatients are admitted independently and are not referred by gatekeepers (cf. [13]). It is recommended that psychiatric healthcare centres offer some kind of advertisement or public relations campaign for their patients (*perspective of recipients*).

## Supplementary Information

Supplementary Material 1.

## Data Availability

The datasets generated and/or analyzed during the current study are not publicly available due to confidentiality being guaranteed to participants but are available from the corresponding author upon reasonable request.

## Abbreviations

EBP: Evidence-based practice
CHIMPS-NET: Children of mentally ill parents network
PRISM: Practical, Robust Implementation and Sustainability Model
ci-chimps: The clinical implementation study of *CHIMPS-NET* with three implementation interventions
CG: Control group
IG: Intervention group
SPSS: Statistical Package for the Social Sciences
Psych: Profession group specialists in psychiatry-neurology-psychosomatics-psychotherapy
Soma: Profession group specialists in family doctor/general practitioner/internist/practicing physician
