# Supplementary material for: Outpatient referrers as a pathway to care for a new family-centered health intervention in psychiatric clinics and how to reach them: evaluation of an implementation strategy
Source: BMC Health Serv Res. 2025 Jul 1;25:866. doi: 10.1186/s12913-025-13031-x (PMC12220561; doi:10.1186/s12913-025-13031-x)
Supplement: Supplementary file 1 — Supplementary Material 1. [file 12913_2025_13031_MOESM1_ESM.docx]

**Criteria for hospital referrals (gatekeeper)**

1a) What significance do the following patient- and illness-related criteria have for your prescription of psychiatric hospital treatment?

very unimportant very important none

Specification

Severity of the current symptoms
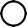

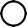

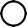

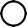

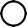

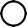

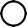


Professional situation
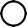

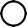

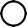

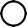

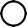

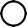

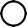


Medical history
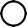

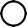

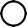

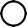

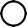

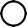

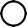


Minor children in the household
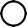

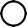

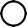

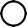

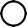

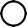

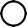


Course of the disease to date
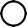

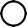

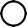

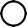

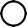

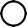

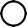


Marital status
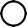

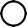

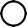

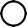

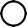

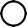

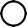


Patient request for inpatient treatment
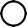

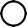

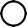

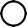

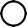

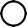

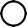


Age of the patient
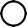

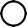

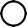

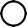

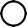

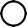

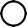


1b) How do you weigh the possible allocation criteria against each other?

*Please draw a line to the box corresponding to the rank you selected (1 = the most important criterion; 8 = the least important criterion).*

| 1  *(the most important criterion)* |
| --- |
| 2 |
| 3 |
| 4 |
| 5 |
| 6 |
| 7 |
| 8  *(the least important criterion)* |

| Severity of the  Symptomatology |
| --- |
| Professional situation |
| Medical history |
| Minors  Children in the household |
| Previous  Course of the disease |
| Marital status |
| Patient request for inpatient treatment  Treatment |
| Age of the patient |


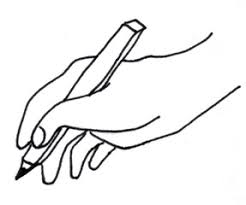


1c) Please estimate the proportion of adult psychiatric patients with a minor child living in the household (prevalence in Germany).

*In percent*

| 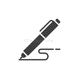 |
| --- |

**Referral to (specialist clinic in the area)**

2a) I am currently admitted to adult psychiatry (specialist clinic in the area).

- Yes
- no

If so, how many patients are involved on average per year?


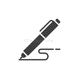


- not specified

2b) Are you aware of any in-house services for parents with underage children in the adult psychiatry department in (specialist clinic in area)?

*For example: Parent-child treatments, parent counseling or groups?*

- yes, namely:
- no

2c) Was the family intervention you mentioned ever a reason for admission?

- Yes
- no

**Sociodemographics**

3a) Please indicate your gender

- female
- Male
- diverse

3b) Please state your age:


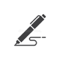


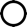
 not specified

3c) Please state your current professional activity:

- Psychiatrist / Specialist in psychiatry / Specialist in psychiatry and psychotherapy
- Neurologist / Specialist in neurology / Specialist in neurology and psychiatry
- Neurology / Specialist in neurology
- Family doctor / General practitioner / General practitioner for internal medicine
- Internist (general practitioner/specialist) / specialist in psychotherapeutic medicine
- Psychological psychotherapist
- Specialist in psychosomatic medicine and psychotherapy
- not specified

3d) Please state the duration of your professional activity in years


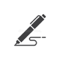


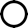
not specified

3e) Please indicate the type of your practice

- Individual practice
- Joint practice
- Medical care center (MVZ)
- not specified

3f) I only treat...

- Patients with statutory health insurance
- Privately insured patients
- both and
- not specified
